# Supplementary figures and images for: Molecular basis of hemoglobin adaptation in the high-flying bar-headed goose
Source: PLoS Genet. 2018 Apr 2;14(4):e1007331. doi: 10.1371/journal.pgen.1007331 (PMC5903655; doi:10.1371/journal.pgen.1007331)

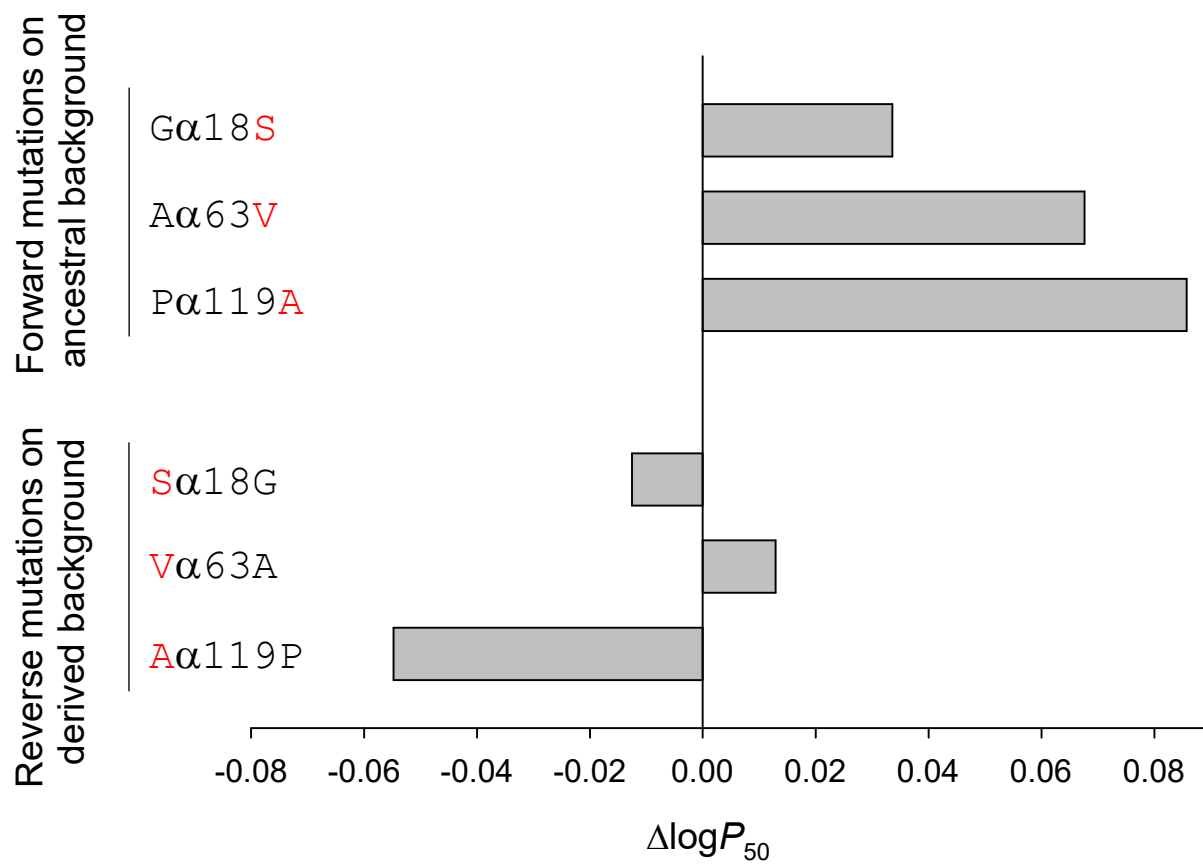

Supplement: S1 Fig — The affinity-enhancing effect of αP119A on the AncAnser background is mirrored by a similarly pronounced affinity-reducing effect when the mutation is reverted on the wildtype bar-headed goose background (αA119P). By contrast, forward and reverse mutations at α18 and α63 do not show the same symmetry of effect, indicating that their effects are conditional on the amino acid state at one or both of the other two sites. (PDF) [file pgen.1007331.s001.pdf]

**A**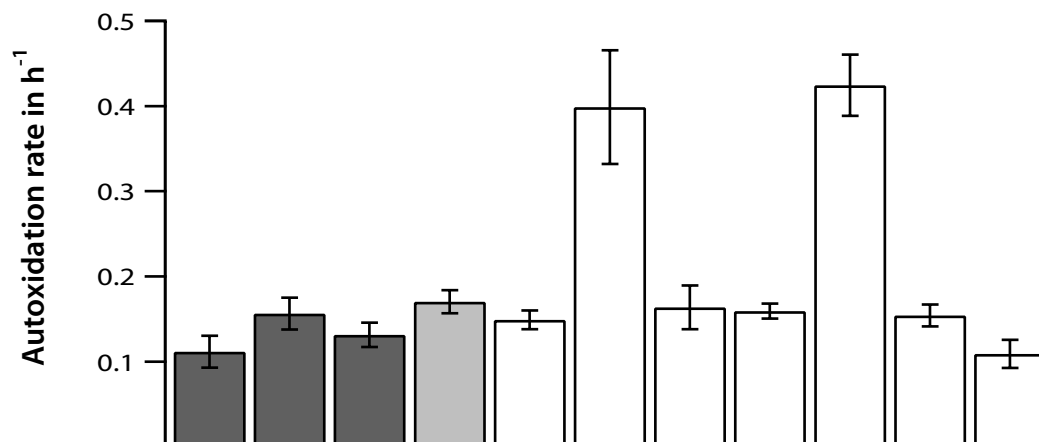**B**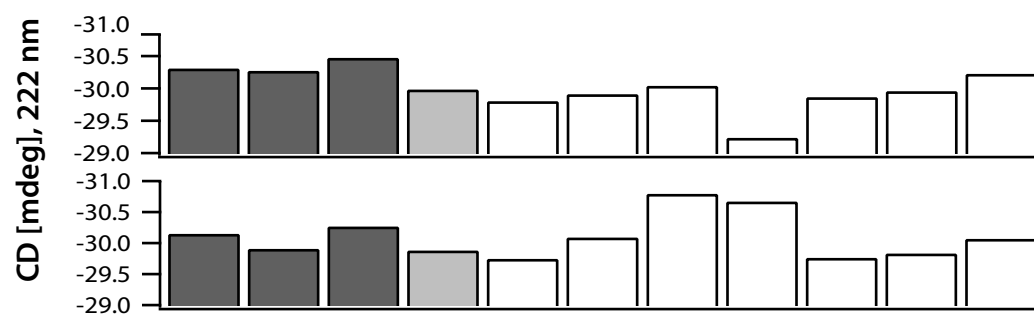**C**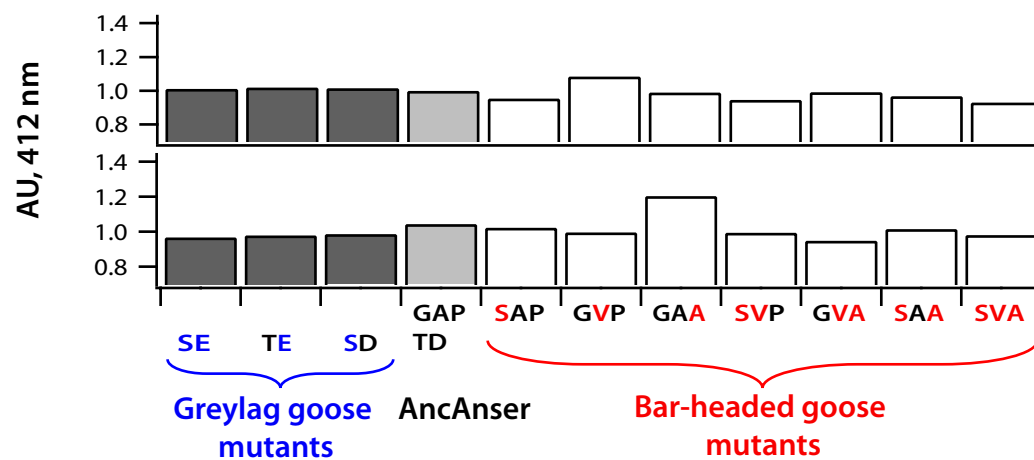

Supplement: S2 Fig — Variation in (A) autoxidation rate (rate at which ferrous heme [Fe2+] spontaneously oxidizes to the ferric state [Fe3+]), (B) secondary structure content, as assessed by means of circular dichroism spectra (with ellipticity measured in millidegrees [mdeg], 222 nm) at pH 7.0 and 7.5 (physiological range), and (C) stability of tertiary structure and holoprotein, as assessed by means of UV-visible spectroscopy (absorbance measured at 412 nm) at pH 7.0 and 7.5 (physiological range). For stability measurements over the full pH range, see S1 and S2 Tables. (PDF) [file pgen.1007331.s002.pdf]

A

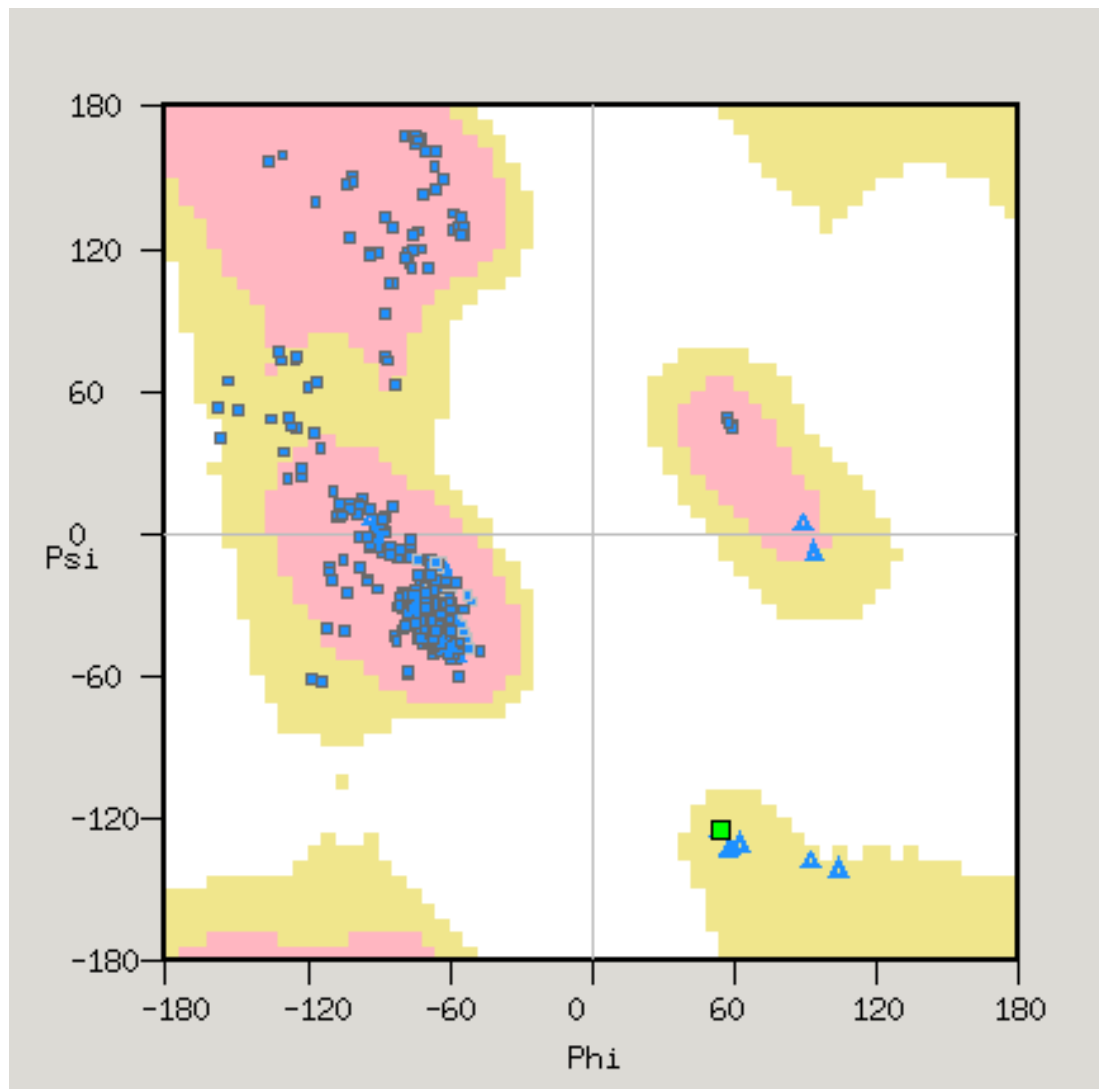

B

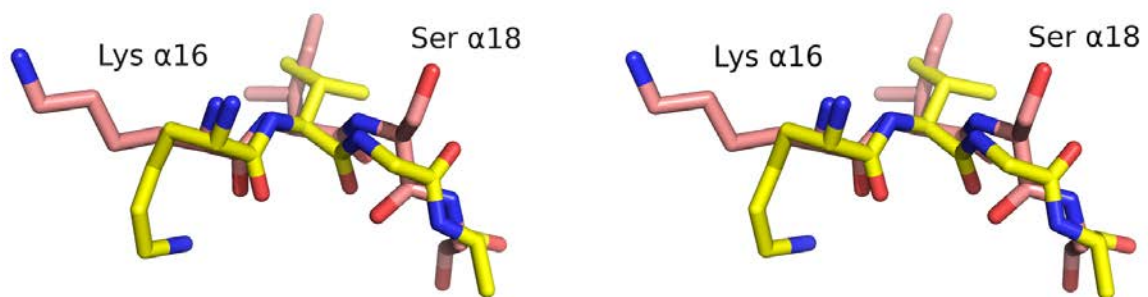

Supplement: S3 Fig — Glycine residues are denoted by triangles, other residues by squares. One residue, α18-Ser, is conspicuous by its unusual backbone angles, and is shown as a green square. This position in the Ramachandran plot is highly unusual for any residue other than glycine. The turn in the backbone between the A and B helices can only be accommodated by a glycine, since the lack of a side-chain avoids the strong steric clash that would develop between a Cβ atom and the nitrogen atom of residue 19. The serine at α18 is therefore forced to flip the peptide conformation, such that its carbonyl group points in the opposite direction relative to that of Gly 18. (PDF) [file pgen.1007331.s003.pdf]
