# Supplementary material for: Molecular basis of hemoglobin adaptation in the high-flying bar-headed goose
Source: PLoS Genet. 2018 Apr 2;14(4):e1007331. doi: 10.1371/journal.pgen.1007331 (PMC5903655; doi:10.1371/journal.pgen.1007331)
Supplement: S1 Table — Measurements of absorbance maxima at 412 nm are shown for rHbs representing wildtype genotypes of bar-headed goose (BHG), greylag goose (GG), their reconstructed ancestor (AncAnser), and all possible mutational intermediates connecting AncAnser with each of the two descendant species. For the bar-headed goose mutants (all mutational intermediates between wildtype bar-headed goose and AncAnser), three-letter genotype codes denote amino acid states at α18, α63, and α119 (amino acid abbreviations in black lettering = ancestral, red lettering = derived). At these same three sites, AncAnser is ‘GAP’ the wildtype genotype of bar-headed goose is ‘SVA’. For the greylag goose mutants (all mutational intermediates between wildtype greylag goose and AncAnser), two-letter genotype codes denote amino acid states at β4 and β125 (amino acid abbreviations in black lettering = ancestral, blue lettering = derived). At these same two sites, AncAnser is ‘TD’ the wildtype genotype of greylag goose is ‘SE’. (DOCX) [file pgen.1007331.s004.docx]

**Table S1.** Effect of pH on the stability of tertiary structure, as measured by UV-visible spectroscopy. Measurements of absorbance maxima at 412 nm are shown for rHbs representing wildtype genotypes of bar-headed goose (BHG), greylag goose (GG), their reconstructed ancestor (AncAnser), and all possible mutational intermediates connecting AncAnser with each of the two descendant species. For the bar-headed goose mutants (all mutational intermediates between wildtype bar-headed goose and AncAnser), three-letter genotype codes denote amino acid states at α18, α63, and α119 (amino acid abbreviations in black lettering = ancestral, red lettering = derived). At these same three sites, AncAnser is ‘GAP’ the wildtype genotype of bar-headed goose is ‘SVA’. For the greylag goose mutants (all mutational intermediates between wildtype greylag goose and AncAnser), two-letter genotype codes denote amino acid states at β4 and β125 (amino acid abbreviations in black lettering = ancestral, blue lettering = derived). At these same two sites, AncAnser is ‘TD’ the wildtype genotype of greylag goose is ‘SE’.

AU, 412nm

| pH | BHG (wt) | GG (wt) | AncAnser | GAA | GVP | SAP | GVA | SAA | SVP | SD | TE |
| --- | --- | --- | --- | --- | --- | --- | --- | --- | --- | --- | --- |
| 2.0 | 0.00 | 0.01 | 0.03 | 0.03 | 0.01 | 0.00 | 0.00 | 0.08 | 0.05 | 0.02 | 0.04 |
| 2.5 | 0.01 | 0.02 | 0.00 | 0.00 | 0.00 | 0.00 | 0.06 | 0.00 | 0.00 | 0.01 | 0.01 |
| 3.0 | 0.05 | 0.12 | 0.05 | 0.05 | 0.07 | 0.03 | 0.12 | 0.04 | 0.03 | 0.12 | 0.11 |
| 3.5 | 0.23 | 0.44 | 0.30 | 0.29 | 0.33 | 0.20 | 0.25 | 0.18 | 0.24 | 0.64 | 0.48 |
| 4.0 | 0.55 | 0.76 | 0.61 | 0.56 | 0.64 | 0.50 | 0.50 | 0.41 | 0.59 | 0.88 | 0.77 |
| 4.5 | 0.73 | 1.01 | 0.78 | 0.82 | 0.89 | 0.77 | 0.74 | 0.69 | 0.79 | 1.08 | 0.99 |
| 5.0 | 0.82 | 1.05 | 0.82 | 0.88 | 1.01 | 0.90 | 0.84 | 0.84 | 0.81 | 1.10 | 1.02 |
| 5.5 | 0.87 | 1.02 | 0.92 | 0.85 | 1.03 | 0.88 | 0.89 | 0.96 | 0.86 | 1.09 | 1.00 |
| 6.0 | 0.90 | 1.04 | 0.94 | 0.86 | 1.08 | 0.88 | 0.91 | 0.95 | 0.95 | 1.09 | 1.02 |
| 6.5 | 0.93 | 1.01 | 1.00 | 0.99 | 1.08 | 0.95 | 0.99 | 0.97 | 0.95 | 1.02 | 1.02 |
| 7.0 | 1.00 | 1.00 | 1.00 | 1.00 | 1.00 | 1.00 | 1.00 | 1.00 | 1.00 | 1.00 | 1.00 |
| 7.5 | 0.98 | 0.97 | 1.04 | 1.20 | 0.99 | 1.02 | 0.95 | 1.02 | 0.99 | 0.99 | 0.98 |
| 8.0 | 1.07 | 1.03 | 1.06 | 1.33 | 1.11 | 1.03 | 1.00 | 1.02 | 0.99 | 1.04 | 0.98 |
| 8.5 | 1.11 | 0.95 | 1.04 | 1.33 | 1.19 | 1.15 | 0.96 | 0.98 | 0.99 | 0.93 | 0.99 |
| 9.0 | 1.14 | 1.01 | 1.08 | 1.43 | 1.33 | 1.23 | 1.08 | 1.02 | 1.06 | 0.98 | 1.00 |
| 9.5 | 1.26 | 0.92 | 1.11 | 1.43 | 1.31 | 1.28 | 1.17 | 1.10 | 1.14 | 0.93 | 0.92 |
| 10.0 | 1.35 | 0.87 | 1.11 | 1.43 | 1.28 | 1.25 | 1.18 | 1.11 | 1.16 | 0.85 | 0.87 |
| 10.5 | 1.40 | 0.80 | 1.08 | 1.43 | 1.23 | 1.18 | 1.12 | 1.05 | 1.09 | 0.73 | 0.79 |
| 11.0 | 1.42 | 0.83 | 1.09 | 1.35 | 1.38 | 1.29 | 1.15 | 1.13 | 1.16 | 0.81 | 0.85 |
