# Supplementary material for: Molecular basis of hemoglobin adaptation in the high-flying bar-headed goose
Source: PLoS Genet. 2018 Apr 2;14(4):e1007331. doi: 10.1371/journal.pgen.1007331 (PMC5903655; doi:10.1371/journal.pgen.1007331)
Supplement: S2 Table — Measurements of molar ellipticity are shown for rHbs representing wildtype genotypes of bar-headed goose (BHG), greylag goose (GG), their reconstructed ancestor (AncAnser), and all possible mutational intermediates connecting AncAnser with each of the two descendant species. For the bar-headed goose mutants (all mutational intermediates between wildtype bar-headed goose and AncAnser), three-letter genotype codes denote amino acid states at α18, α63, and α119 (amino acid abbreviations in black lettering = ancestral, red lettering = derived). At these same three sites, AncAnser is ‘GAP’ the wildtype genotype of bar-headed goose is ‘SVA’. For the greylag goose mutants (all mutational intermediates between wildtype greylag goose and AncAnser), two-letter genotype codes denote amino acid states at β4 and β125 (amino acid abbreviations in black lettering = ancestral, blue lettering = derived). At these same two sites, AncAnser is ‘TD’ the wildtype genotype of greylag goose is ‘SE’. (DOCX) [file pgen.1007331.s005.docx]

**Table S2.** Stability of α-helical secondary structure as a function of pH, measured by circular dichroism spectroscopy. Measurements of molar ellipticity are shown for rHbs representing wildtype genotypes of bar-headed goose (BHG), greylag goose (GG), their reconstructed ancestor (AncAnser), and all possible mutational intermediates connecting AncAnser with each of the two descendant species. For the bar-headed goose mutants (all mutational intermediates between wildtype bar-headed goose and AncAnser), three-letter genotype codes denote amino acid states at α18, α63, and α119 (amino acid abbreviations in black lettering = ancestral, red lettering = derived). At these same three sites, AncAnser is ‘GAP’ the wildtype genotype of bar-headed goose is ‘SVA’. For the greylag goose mutants (all mutational intermediates between wildtype greylag goose and AncAnser), two-letter genotype codes denote amino acid states at β4 and β125 (amino acid abbreviations in black lettering = ancestral, blue lettering = derived). At these same two sites, AncAnser is ‘TD’ the wildtype genotype of greylag goose is ‘SE’.

CD [mdeg], 222nm

| pH | BHG (wt) | GG (wt) | AncAnser | GAA | GVP | SAP | GVA | SAA | SVP | SD | TE |
| --- | --- | --- | --- | --- | --- | --- | --- | --- | --- | --- | --- |
| 2.0 | -13.36 | -11.29 | -11.75 | -11.16 | -12.17 | -11.61 | -10.77 | -13.90 | -13.31 | -9.10 | -7.96 |
| 2.5 | -13.75 | -10.64 | -11.79 | -9.87 | -13.01 | -11.61 | -11.14 | -11.51 | -12.35 | -8.60 | -8.21 |
| 3.0 | -14.85 | -12.09 | -11.86 | -9.11 | -13.33 | -12.59 | -11.41 | -12.08 | -12.43 | -8.75 | -8.68 |
| 3.5 | -18.13 | -17.61 | -14.86 | -11.70 | -17.35 | -15.36 | -15.01 | -15.08 | -18.02 | -19.56 | -14.89 |
| 4.0 | -24.60 | -22.50 | -23.12 | -19.81 | -24.70 | -22.75 | -22.87 | -21.13 | -24.33 | -23.43 | -21.55 |
| 4.5 | -28.04 | -27.45 | -27.45 | -26.11 | -28.47 | -27.61 | -29.51 | -27.08 | -28.12 | -27.34 | -26.86 |
| 5.0 | -30.00 | -29.83 | -28.95 | -28.52 | -29.20 | -28.94 | -29.28 | -28.68 | -28.67 | -29.52 | -28.66 |
| 5.5 | -30.30 | -30.53 | -29.07 | -29.24 | -28.85 | -29.75 | -29.77 | -29.02 | -28.81 | -30.55 | -29.86 |
| 6.0 | -29.41 | -30.95 | -29.81 | -30.59 | -29.06 | -30.25 | -29.81 | -29.40 | -29.45 | -30.72 | -30.05 |
| 6.5 | -30.23 | -30.31 | -29.98 | -30.04 | -29.91 | -29.80 | -29.87 | -29.96 | -29.24 | -30.47 | -30.28 |
| 7.0 | -30.00 | -30.00 | -30.00 | -30.00 | -30.00 | -30.00 | -30.00 | -30.00 | -30.00 | -30.00 | -30.00 |
| 7.5 | -30.07 | -30.15 | -29.88 | -30.79 | -30.09 | -29.75 | -29.76 | -29.83 | -30.67 | -30.27 | -29.91 |
| 8.0 | -30.07 | -30.18 | -29.74 | -30.76 | -30.37 | -30.03 | -29.52 | -29.46 | -30.50 | -29.24 | -29.71 |
| 8.5 | -29.56 | -30.29 | -29.76 | -30.76 | -29.73 | -30.26 | -30.32 | -29.94 | -30.96 | -29.32 | -28.93 |
| 9.0 | -30.09 | -29.53 | -30.11 | -31.29 | -30.14 | -29.74 | -30.21 | -30.24 | -30.98 | -30.19 | -29.27 |
| 9.5 | -30.42 | -30.93 | -30.18 | -31.29 | -30.28 | -29.87 | -30.72 | -30.16 | -31.37 | -29.09 | -29.28 |
| 10.0 | -30.30 | -30.25 | -29.79 | -31.19 | -29.87 | -29.96 | -30.21 | -30.08 | -31.32 | -29.65 | -28.85 |
| 10.5 | -28.92 | -28.82 | -29.30 | -31.19 | -29.25 | -29.40 | -29.08 | -29.27 | -30.20 | -28.97 | -27.63 |
| 11.0 | -28.70 | -29.77 | -29.58 | -30.10 | -28.98 | -29.55 | -29.65 | -29.69 | -30.28 | -29.46 | -28.19 |
